# Lessons learned from a periodontal intervention to reduce progression of chronic kidney disease among Aboriginal Australians

**DOI:** 10.1186/s13104-020-05317-6

**Published:** 2020-10-15

**Authors:** Lisa M. Jamieson, Cherian Sajiv, Alan Cass, Louise J. Maple-Brown, Michael R. Skilton, Kostas Kapellas, Basant Pawar, Peter Arrow, Lisa M. Askie, Wendy Hoy, David Harris, Alex Brown, Jaquelyne T. Hughes

**Affiliations:** 1 Australian Research Centre for Population Oral Health, University of Adelaide Dental School, University of Adelaide, Adelaide, SA Australia; 2 Central Australian Renal Services, Northern Territory Department of Health, Darwin, NT Australia; 3 Menzies School of Health Research, Charles Darwin University, Casuarina, NT Australia; 4 Boden Collaboration for Obesity, Nutrition, Exercise and Eating Disorders, Faculty of Medicine and Health, The University of Sydney, Sydney, NSW Australia; 5 Western Australian Dental Services, Western Australian Government, Perth, WA Australia; 6 NHMRC Clinical, Trials Centre, University of Sydney, Sydney, NSW Australia; 7 Centre for Chronic Disease, University of Queensland, Brisbane, QLD Australia; 8 Westmead Centre for Medical Research, University of Sydney & Department of Renal Medicine, Westmead Hospital, Sydney, NSW Australia; 9 Aboriginal Research Unit, South Australian Health & Medical Research Institute, Adelaide, SA Australia; 10 Department of Nephrology, Division of Medicine, Royal Darwin Hospital, Darwin, NT Australia

**Keywords:** Periodontal disease, Chronic kidney disease, Aboriginal Australian

## Abstract

**Objective:**

Periodontal disease is associated with chronic kidney disease (CKD), with both conditions being highly prevalent among Australia’s Aboriginal population. This paper reflects on the lessons learned following implementation of a periodontal intervention in the Central Australian region of the Northern Territory among Aboriginal adults with CKD.

**Results:**

Between Oct 2016 and May 2019, research staff recruited 102 eligible participants. This was far below the anticipated recruitment rate. The challenges faced, and lessons learned, were conceptualised into five specific domains. These included: (1) insufficient engagement with the Aboriginal community and Aboriginal community-controlled organisations; (2) an under-appreciation of the existing and competing patient commitments with respect to general health and wellbeing, and medical treatment to enable all study commitments; (3) most study staff employed from outside the region; (4) potential participants not having the required number of teeth; (5) invasive intervention that involved travel to, and time at, a dental clinic. A more feasible research model, which addresses the divergent needs of participants, communities and service partners is required. This type of approach, with sufficient time and resourcing to ensure ongoing engagement, partnership and collaboration in co-design throughout the conduct of research, challenges current models of competitive, national research funding.

## Introduction

Aboriginal Australians experience high rates of chronic kidney disease (CKD) [1]. Recent estimates from Central Australia suggest that more than 40 percent of Aboriginal Health Service attendees have micro or macroalbuminuria and 40 percent have reduced eGFR [2]. Dialysis for end stage kidney disease (ESKD) is the leading cause of hospitalisation for Aboriginal Australians, comprising 42 percent of all admissions [3]. Aboriginal Australians with ESKD are younger than their non-Aboriginal counterparts and many have co-existing chronic health conditions [4].

Periodontal disease is inflammation of the tissues surrounding teeth and results from a complex interplay between bacteria and host risk factors. Periodontal organisms flourish in the presence of inflammation, enabling them to invade host tissues and to gain direct access to the circulation [5]. The constant exposure of the vasculature to periodontal pathogens provides an opportunity for endothelial inflammatory activation and functional impairment [6]. Clinically, this manifests as deepening of the epithelial attachment around teeth, loss of periodontal attachment, tooth loosening and, ultimately, tooth loss. Biological plausibility for considering periodontal disease as a CKD risk factor is derived from the potential role of the inflammatory response to periodontal disease in the chronic systemic inflammatory burden associated with CKD [7]. Specifically, the local tissue destructive immuno-inflammatory response to periodontal pathogens, their products and inflammatory cytokines are believed to contribute to the chronic systemic inflammatory burden [8].

We recently conducted a randomised controlled trial (RCT) that aimed to reduce surrogate markers of cardiovascular disease through a periodontal intervention in an otherwise healthy adult cohort of Aboriginal Australians in the Northern Territory who had moderate or severe periodontal disease (the PerioCardio study) [9]. Based on the evidence from the PerioCardio study, we were approached by key kidney and dental health stakeholder groups in Central Australia to build a research partnership with the overall goal being to implement a RCT to improve the oral health of adults requiring dialysis in this location [10]. In this paper we describe the successes and challenges of the methodology, and propose alternate approaches to concurrently achieve improved periodontal health in Central Australia for adults living with CKD and ESKD.

## Main text

### Methods

Sample size calculations estimated that 240 participants would be necessary at baseline. To be eligible, participants needed to: (1) identify as being Aboriginal or Torres Strait Islander Australian; (2) be aged 18+ years; (3) have CKD; (4) have moderate or severe periodontal disease; (5) have at least 12 teeth (later amended to 8 teeth) and; (6) reside in Central Australia. The periodontal intervention was based on Tonetti et al. recommended technique, which involves intensive removal of subgingival dental plaque biofilms by scaling, root-planing and removal of teeth that cannot be saved, following administration of local anaesthesia [11]. Ethical approval was obtained from the University of Adelaide Human Research Ethics Committee and the Central Australian Human Research Ethics Committee. Written informed consent was obtained from study participants.

Study feasibility was dependent on partnership between the research team and the Central Australian primary and tertiary health care sector. Specific recruitment strategies included: establishing initial service agreements with the key Aboriginal community-controlled health and government health services; partnering with community champions, some of whom were previously involved with projects with the study investigators; engaging with awareness raising activities (for example, the role of periodontal disease in systemic health) to promote the opportunity to participate with key community stakeholder groups and; presentations made to community groups. As there were wide inclusion criteria for the study (diabetes or any indicator of CKD), a passive snowballing approach was the preferred technique with participants encouraged to inform friends, family and peers of ways to contact the research team to register interest in partaking. An Indigenous study reference group was established prior to commencement of recruitment, to guide processes and to act as champions for the study in the community. The retention strategies utilised involved: (1) employing Aboriginal people in roles to undertake contact tracing to enhance participant follow-up at scheduled study visits; (2) ensuring participants were contacted on a regular basis to check accuracy of contact details; (3) maintaining relationships with appropriate stake holders whose role was to promote the study; (4) ascertaining contact details of three key personnel who may know the whereabouts of participants should the study team be unable to contact them; (5) sending birthday and Christmas cards to participants and; (6) supporting continuity of care in the research conduct between staff and participants where desired (by participants), with study staff ideally seeing each of their participants for each phase of the research [12].

Across the course of the project there were fourteen staff employed in participant recruitment roles. The professional skill-set of employed research staff included dentistry, social work, psychology and Aboriginal public health research. Four of the research staff were Aboriginal people, which was a strong preference of the study design [13]. Prior to data collection, all non-Aboriginal project staff completed relevant Aboriginal cultural competency training. Collectively, the 14 research staff held many person-years’ experience working with and engaging Aboriginal communities in a range of health research projects. The on-ground research staff were supported by a study investigator team with expertise in Aboriginal health, oral health, kidney disease and epidemiology, including 50% who were local residents of the study region, and research governance processes including a Study Management Group. The Study Management Group provided oversight and general leadership of the study, including strategies for staff recruitment, participant recruitment and liaisons with key stakeholder groups.

Qualitative records summarising research staff contact with potential participants and key stakeholder groups were collected throughout the study. These records, together with field trip notes, researcher reflections, and feedback from individuals and organisations involved in the study were reviewed to summarise the main challenges in implementing the trial.

### Results

From Oct 2016 to May 2019, 102 participants were enrolled; far below the anticipated recruitment rate. There were no serious adverse events reported. After analysing data collected from the qualitative evaluation processes of the study, five key challenges faced, and lessons learned, in operationalising the proposed trial emerged. These are outlined below and in Table 1.

**Table 1:** Summary of challenges experienced and major learnings in a periodontal intervention among Aboriginal Australians with kidney disease in Central Australia

Challenges experienced	Major learnings
Insufficient engagement with the Aboriginal community and Aboriginal community-controlled organisations	Engage with Aboriginal Australians with kidney disease and their ACCHO to ascertain if periodontal and oral health care is a priority If yes, work in partnership to derive feasible study aims, objectives and design, ensuring the research methodologies involved will be culturally acceptable Involve those with lived experience of kidney disease to be both investigators on subsequent study grant applications and involved with the study’s consumer representative group/Aboriginal reference group Facilitate regular Aboriginal reference group meetings, being aware of competing demands and expectations on reference group members Engage in regular knowledge exchange forums with all stakeholder groups; Aboriginal Australians with kidney disease, ACCHOs, and health provider stakeholders Facilitate opportunities for Aboriginal Australians with kidney disease, ACCHOs and health provider stakeholders to be involved in data interpretation, analysis, write-ups for publication and presentation at local, national and international conferences
Co-ordinating around patient commitments, general health and wellbeing, and medical treatment	Consider if, in light of the many comorbidities and social vulnerabilities experienced by those eligible, a periodontal intervention requiring multiple visits for dental care is, in fact, feasible If it is considered to be feasible, carefully take into account the general health and well-being, patient commitments and medical care required (especially for dialysis) and the logistical planning required to ensure the study objectives can be met without compromising participant health and wellbeing It may be more practical/ethical to spread out the study recruitment and treatment phases so that the impost of participants re: dental care is reduced
Study staff not primarily from the Northern Territory	Consider conducting the study in the state or regional location in which the CIA is located (in this case, South Australia) Consider increasing the number of study sites to involve multiple jurisdictions, and have the study team based in the CIA’s location operate on a ‘fly in/fly out’ model Ensure the Aboriginal Reference Group is involved in selection of study staff and that appropriate training in Aboriginal cultural competency is provided
Potential participants not having the required number of teeth	Co-ordinate with local public dental health providers and/or dentists employed by local ACCHOs to ascertain, broadly, the anticipated number of teeth among Aboriginal Australians with kidney disease Consult the literature/periodontal disease experts to ascertain if a threshold of less than 8 teeth might in fact be reasonable for a periodontal intervention involving Aboriginal Australians with kidney disease
Invasive intervention that involved travel to, and time at, a dental clinic	As much as can be accommodated, plan for the provision of dental care to take place in the minimal time possible Ascertain with each participant their preferred time of day/day of week for dental care and how long they can realistically spend at each dental visit If possible, work with providers of dental care facilities to enable dental care to study participants on weekends If access to dental vans is possible, consider this as an option to move the dental clinic closer to participant locations

1. *Insufficient engagement with the Aboriginal community and Aboriginal community*-*controlled organisations*, specifically, insufficient engagement in setting research priorities followed by designing approaches to address them. Prior to writing the grant application, and the subsequent receipt of funding, the engagement had been done primarily with the stakeholder groups providing kidney care to Aboriginal Australians with kidney disease and dental health service providers in Central Australia. Only a small proportion of the engagement involved Aboriginal Australians with kidney disease themselves or their primary health care organisations, usually the local Aboriginal community-controlled health organisation (ACCHO). At study commencement, a letter of support was provided by the main ACCHO, but the study design had already been decided upon. We did have an Aboriginal cultural ambassador at the study’s commencement, who did facilitate one Aboriginal Reference Group meeting with the CKD patient group in Central Australia (which resulted in employment of an Aboriginal research officer), but the early departure of the study’s first project manager resulted in a lull in study operations. There were no further Aboriginal Reference Group meetings when the study recommenced. This reflected a break-down in handover of this crucial governance and leadership process with the Aboriginal Reference Group.

2. *Co*-*ordinating around patient commitments, general health and wellbeing, and medical treatment* To be eligible for the study, participants needed to have an indicator of CKD. However, early stage kidney disease is often asymptomatic and not necessarily known to individuals or identified by their health care providers [14]. It was not a function of the study to screen the general public with a random urine ACR test. Participants with established and advanced CKD requiring dialysis (around 84 percent of the sample) required a crucial health care commitment of 4–5 h dialysis treatments three times a week with additional burden of travel time to treatment. The additional 3 h of study assessment greatly impacted the logistical ability of potential participants to be involved, who also needed to attend to personal care, care for other health conditions, and family and cultural responsibilities.

3. *Study staff not primarily from the Northern Territory.* Fourteen staff were employed over the course of the project. This indicated a desire by industry personnel to contribute to improved oral health in this region. However, as most staff (n = 11) were recruited from cities in other states and territories, it also reflected difficulties in accessing a skilled workforce for all required study duties in the local area. We recognise the emerging priority of skilled Indigenous workforce to deliver health care for Aboriginal and Torres Strait Islander people. This is reflected in national policy, local workforce, and Aboriginal peoples. Relationships are essential in health research involving Aboriginal Australians, so project staff from outside the Northern Territory faced huge additional barriers which frequently resulted in them terminating their contracts early. The reasons for this included: (i) geographic and social isolation of relocating to Central Australia; (ii) frustration with recruitment difficulties; (iii) ill health of family members and; (iv) other employment/higher education opportunities.

4. *Potential participants not having the required number of teeth.* In order to have periodontal disease, a participant needed to have teeth. Although there is no threshold number of teeth required for periodontal interventions, the standard premise is 2–3 per quadrant of the mouth. The initial 12 teeth thus related to, on average, 3 teeth per quadrant, with the revised 8 teeth roughly relating to 2 teeth per quadrant. Forty-five potential participants were not eligible because they had fewer than 8 teeth. This included 22 potential participants who were edentulous (no teeth). Because there were no estimates available on the number of teeth of Aboriginal Australians with kidney disease in Central Australia, this was a recruitment challenge the study team had not anticipated.

5. *Invasive intervention that involved travel to, and time at, a dental clinic.* The intervention required participants to travel to, and spend time at, a dental clinic to receive the required periodontal care, sometimes in two or more visits. These visits were facilitated by research staff, and were free, but did require careful logistics regarding dialysis visits and other medical priorities. In this highly vulnerable population, it was frequently not realistic to schedule dental visits when there were so many other competing health and family concerns. A number of participants had never attended for dental care before, and many were apprehensive or scared of receiving care.

There were many strategies employed to try and mitigate the ongoing challenges faced in the recruitment of this study. Ultimately, however, two participating ACCHOs withdrew, concerned that the study was not feasible. Because the yield of study participants was not sufficient to justify continuation of the trial as planned, it was terminated prematurely. It invites the question as to why the PerioCardio study was successful when the current study was not. The only difference was that participants in the current study needed to have CKD, with a large proportion being on dialysis. We had not fully anticipated the additional challenges that having CKD/being on dialysis would bring to bear on the study, including the crucial partnerships with primary and tertiary health care providers.

## Limitations

Our study proposed an important methodology, with a number of previously-tested methods and developed stakeholder relationships that suggested the study could be delivered in order to test the value of the intervention. However, in spite of top-level kidney and dental stakeholder support, the large potentially eligible denominator population in the recruitment region and active engagement of the study team, the required number of referrals/recruitment was insufficient. In reflecting on the study design, we acknowledge the greatest reason for the study challenges was likely that, although addressing a priority of kidney health and dental stakeholders, it did not necessarily address an identified priority of Aboriginal communities and ACCHOs. Future intervention studies may be more successful if Aboriginal communities and ACCHOs identify oral health as a priority for those with kidney disease and are engaged in all aspects of the study design, recruitment and implementation phases. It may be advantageous if multiple partners and sites are considered, with sufficient lead-up to enable time for thorough and deep consultation, leading to strong engagement and evidence of ongoing approval from all relevant health service and community stakeholders.

## Data Availability

Available from corresponding author upon request

## Abbreviations

ACCHO: Aboriginal Community Controlled Health Organisation
ACR: Albumin-creatinine ratio
CKD: Chronic kidney disease
eGFR: Estimated glomerular filtration rate
ESKD: End-stage kidney disease
RCT: Randomised controlled trial
